# Genotypes of Pain and Analgesia in a Randomized Trial of Irritable Bowel Syndrome

**DOI:** 10.3389/fpsyt.2022.842030

**Published:** 2022-03-23

**Authors:** Jan Vollert, Ruisheng Wang, Stephanie Regis, Hailey Yetman, Anthony J. Lembo, Ted J. Kaptchuk, Vivian Cheng, Judy Nee, Johanna Iturrino, Joseph Loscalzo, Kathryn T. Hall, Jocelyn A. Silvester

**Affiliations:** ^1^Department of Surgery and Cancer, Faculty of Medicine, Imperial College London, London, United Kingdom; ^2^Department of Neurology, University Hospital of Schleswig-Holstein, Kiel, Germany; ^3^Department of Anesthesiology, Intensive Care and Pain Medicine, University Hospital Muenster, Muenster, Germany; ^4^Mannheim Center of Translational Neuroscience, Medical Faculty Mannheim, Heidelberg University, Heidelberg, Germany; ^5^Department of Medicine, Brigham Women's Hospital, Boston, MA, United States; ^6^Department of Medicine, Harvard Medical School, Boston, MA, United States; ^7^Division of Gastroenterology, Boston Children's Hospital, Boston, MA, United States; ^8^Division of Gastroenterology, Beth Israel Deaconess Medical Center, Boston, MA, United States; ^9^Program in Placebo Studies, Beth Israel Deaconess Medical Center, Boston, MA, United States; ^10^Department of General Medicine Primary Care, Beth Israel Deaconess Medical Center, Boston, MA, United States

**Keywords:** pain, irritable bowel syndrome, genotype, randomized controlled trial, genome-wide association study

## Abstract

**Background:**

Irritable bowel syndrome (IBS) is a highly prevalent chronic pain disorder with multiple underlying mechanisms and few treatments that have been demonstrated to be effective in placebo controlled trials. One potential reason may be the use of composite outcomes, such as the IBS Symptom Severity Scale (IBS-SSS) which includes descriptive items related to pain frequency and pain intensity as well as bowel dysfunction and bloating. We investigated if different features of IBS pain have distinct genetic associations and if these may be moderated by sex hormones.

**Participants and Setting:**

Adult outpatients with moderately severe IBS (>175 on IBS-SSS) enrolled in a clinical trial reported IBS-SSS at baseline and after 6 weeks of therapy.

**Methods:**

Fixed effects modeling was used to test the effect of *COMT* rs4680 genotype to change in pain severity (rated 0-100) and pain frequency (defined as number of days with pain in the past 10 days) from baseline to week 6 with IBS treatment. Parallel exploratory genome-wide association studies (GWAS) were also performed to identify single nucleotide polymorphisms (SNPs) associated with change in pain severity or pain frequency across all participants.

**Results:**

A total of 212 participants (74% female) were included. The *COMT* rs4680 met allele was associated with decreased pain severity over the course of the trial in gene dosage models [beta(SE) −5.9 (2.6), *P* = 0.028]. Exploratory GWAS for change in pain frequency identified 5 SNPs in close proximity on chromosome 18 near *L3MBTL4* which reached genome-wide significance (all *P* < 5.0E-8). This effect was not mediated by changing estradiol levels. There was also a region of chromosome 7 with 24 SNPs of genome-wide suggestive significance for change in pain severity (all *P* < 1.0E-5).

**Conclusions:**

Previously reported association between *COMT* rs4680 genotype and treatment response as measured by IBS-SSS is related to pain severity, but not pain frequency. We also identified new candidate genes associated with changes in IBS pain severity (*SNX13*) and pain frequency (*L3MBTL4*) in response to treatment. Further studies are needed to understand these associations and genetic determinants of different components of IBS-SSS. ClinicalTrials.gov, Identifier: NCT0280224.

## Introduction

Irritable bowel syndrome (IBS) is a chronic functional gastrointestinal disorder characterized by visceral pain, bloating and altered bowel habits (1) that is one of the top 10 reasons for seeing a primary care physician (2, 3). Although visceral pain is a defining characteristic of IBS that takes a considerable toll on quality of life (4), few studies have examined factors that influence a patient's experience of pain. The economic burden of IBS is high and there are few effective treatments, particularly for visceral pain. Furthermore, many common pain drugs have adverse effects that exacerbate IBS symptoms (5).

The identification of drugs that are effective for visceral pain in IBS has been thwarted by high placebo response rates (~40%) in randomized controlled trials (6). Typically, the IBS Severity Scoring System (IBS-SSS) (7) is used to quantify IBS symptoms and assess response to treatment in clinical trials. This validated composite measure includes five items each of which are rated on a 0–100 visual analog scale (abdominal pain, number of days with abdominal pain, severity of abdominal distension, satisfaction with bowel habits, and IBS-related quality of life).

Identification of genetic variants that influence placebo response in IBS could allow for stratification of patients based on their propensity to respond to placebo treatment thereby increasing the precision of clinical trials. Our group identified genes that influenced treatment response to placebo in a randomized trial in which the primary outcome was IBS-SSS (8). Specifically, our group identified that participants with IBS who were homozygous for the catechol-O-methyltransferase (*COMT*) rs4680 met (met/met) had the greatest improvement across treatment arms as measured by the IBS-SSS. However, it remains unclear which of the five items in the IBS-SSS were most significantly influenced by these associations. To date no studies have examined genetic links to changes in the two pain related items in the IBS-SSS (pain severity and pain frequency) which comprise 40% of the composite score in response to IBS treatment. The goals of this study were to determine whether *COMT* variants are linked to the two pain components of IBS-SSS and to identify other genetic variants that influence changes in either pain frequency or pain severity in response to treatment for IBS.

## Methods

This is a *post-hoc*, exploratory analysis of existing genotype data from participants in the Effects of Open-label vs. Double-blind treatment in IBS clinical trial. In this trial, participants were randomized to one of three placebo treatments: open-label placebo (OLP), double-blind placebo (DBP), or no pill control (NPC) (9). To allow for the DBP treatment arm, a small number of participants were randomized to a fourth arm: double-blind peppermint oil (DBM). As described previously, adults who met the Rome IV criteria for IBS with symptoms of moderate or greater severity (defined as a score of ≥175 on the IBS-SSS) were eligible to participate if their IBS medication regimen (e.g., fiber, tricyclic antidepressants, anti-spasmodics, etc.) had been stable for at least 30 days and they agreed not to change their IBS treatment for the duration of the trial (9, 10). Participants were excluded if they reported alarm features, severe acid reflux, use of peppermint oil in the past 30 days, or allergy to soybean oil (used in the placebo pills). The primary outcome was IBS-SSS which was assessed at baseline and at week six by blinded research assistants. OLP and NPC participants knew their treatment assignment. Those assigned to DBP or DBM were told that they were enrolled in an RCT but were not informed of their treatment assignment. Blood was collected for genotyping at the first study visit.

### Genotyping

Genotyping was conducted on the Infinium Global Screening Array v2.0 (Illumina, San Diego, California, US). Quality control of samples was carried out to filter extremely low-quality samples and variants (call rate <97.5%) using PLINK (version 1.9). A total of 729,526 SNPs were mapped to the GRCh37 (hg19) reference genome. To reduce heterogeneity in population structure, we conducted principal component analysis using PLINK on the whole genome SNP data and extracted the top five principal components for correcting genetic heterogeneity across different races/ethnic groups. We limited our analyses to SNPs with minor allele frequency > 0.05 and with a Hardy-Weinberg equilibrium *P* > 1^*^10^−6^.

### Candidate Gene Analysis

A candidate gene analysis was performed using fixed effects models to test the effects of the *COMT* rs4680 genotype (val/val, val/met, or met/met) on the change in pain severity or pain frequency with IBS treatment.


$$\begin{array}{r} ChangePainSev\;\sim \;rs4680\;+\;Age\;+\;Gender\;+\;Treatment \end{array}$$

(1) $$\begin{array}{r} \;+\;PC1\;+\;PC2\;+\;PC3\;+\;PC4\;+\;PC5 \end{array}$$

$$\begin{array}{r} ChangePainFreq\;\sim \;rs4680\;+\;Age\;+\;Gender\;+\;Treatment \end{array}$$

(2) $$\begin{array}{r} \;+\;PC1\;+\;PC2\;+\;PC3\;+\;PC4\;+\;PC5 \end{array}$$

To control for confounding factors, all models included age and sex of the patients, as well as the study arm that the patients were allocated to and the first five principal components identified from the genotype data to correct for genetic heterogeneity across different races/ethnic groups. Principal components analysis (PCA) was performed on the whole genome SNP data using PLINK (11). As sensitivity tests, all analyses were performed for the whole cohort and separately for female participants only to assess signal stability.

### Genome-Wide Association Study

The following models were used for parallel exploratory GWAS on change in pain severity and pain frequency:


$$\begin{array}{r} ChangePainSev\;\sim \;SNP\;+\;Age\;+\;Gender\;+\;Treatment \end{array}$$

(3) $$\begin{array}{r} \;+\;PC1\;+\;PC2\;+\;PC3\;+\;PC4\;+\;PC5\; \end{array}$$

$$\begin{array}{r} ChangePainFreq\;\sim \;SNP\;+\;Age\;+\;Gender\;+\;Treatment \end{array}$$

(4) $$\begin{array}{r} \;+\;PC1\;+\;PC2\;+\;PC3\;+\;PC4\;+\;PC5\; \end{array}$$

In GWAS of quantitative change, the baseline measure has been shown to bias the effect of variants on treatment response; therefore, we did not include baseline painseverity or pain frequency at baseline as a covariate in the model (12). Based on the findings in the exploratory GWAS, we analyzed the effect size [Standardized Mean Difference (SMD)] of hetero- and homozygous variants of the leading SNP of each of the two GWAS. Since a lead finding has been previously linked to pain in dysmenorrhea (13) and IBS symptom fluctuation has been linked to the menstrual cycle (10, 14), we performed a mediation analysis of estradiol on the change in pain frequency linked to the lead SNP of the GWAS. We applied a full mediation model, tested using the PROCESS implementation (15, 16) for IBM SPSS.

## Results

### Patient Cohort

**Figure 1:**
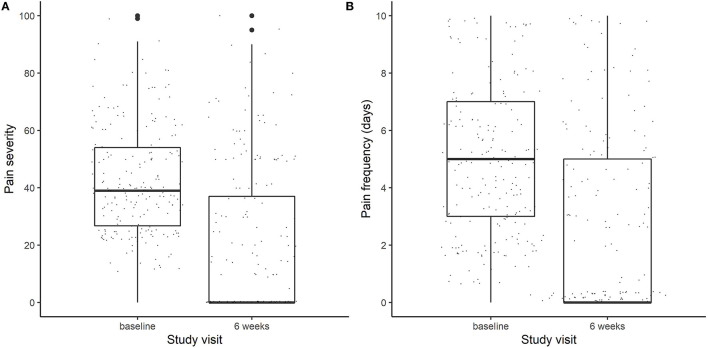
Boxplots of **(A)** pain severity (on a scale of 0–100) and **(B)** Pain frequency (measured in days out of the last 10 days) at baseline and at the end of the trial (week 6).

In this study, we analyzed those participants (*n* = 212) randomized to DBM (*n* = 26), DBP (*n* = 62), OLP (*n* = 62), or NPC (*n* = 62) for whom pain outcomes at baseline and at week 6, as well as genotyping were available. The average age was 42.6 years (74% female) and a majority self-reported their race as white (84%; Table 1). Overall, the average IBS-SSS at baseline was 274.4, with an average pain intensity of 42.7 out of 100 and average pain frequency of 5.1 days over the past 10 days (Figure 1).

**Table 1:** Demographics and change in IBS Symptom Severity Score (IBS-SSS) pain severity and pain frequency components by treatment arm.

	**Double-blind Mint (DBM)**	**Double-blind Placebo (DBP)**	**Open-label Placebo (OLP)**	**No-pill Control (NPC)**
*N*	26	62	62	62
Age, mean (SD)	43.4 (16.9)	43.5 (20.5)	43.1 (17.7)	40.8 (17.5)
Female, (%)	18 (69.2)	46 (74.2)	44 (77.4)	48 (71.0)
White, (%)	22 (84.6)	53 (85.5)	52 (83.9)	52 (83.9)
Change in pain severity, mean (SD)	30.5 (21.6)	23.0 (26.1)	25.7 (25.3)	17.8 (28.3)
Change in pain frequency, mean (SD)	2.3 (3.8)	3.2 (4.0)	2.2 (4.0)	1.4 (3.7)

### COMT Association With Change in Pain Frequency and Pain Severity Items on IBS-SSS

In *COMT* rs4680 gene dosage models of change in the pain frequency and pain severity components of the IBS-SSS from baseline to 6-weeks, increasing number of met alleles was associated with a significantly greater reduction in IBS pain severity [beta(SE), −5.9 (2.6), *P* = 0.028], but not frequency [beta(SE), −0.52(0.40), *P* = 0.198] across all participants combined. Sensitivity analysis with women only revealed a similar pattern of *COMT* rs4680 effects across all treatment arms in females, such that met/met women had the greatest change in pain severity (29.7 ± 25.5) and val/val women the least (17.5 ± 26.6).

### Exploratory GWAS of Analgesic Effects During the Trial

**Figure 2:**
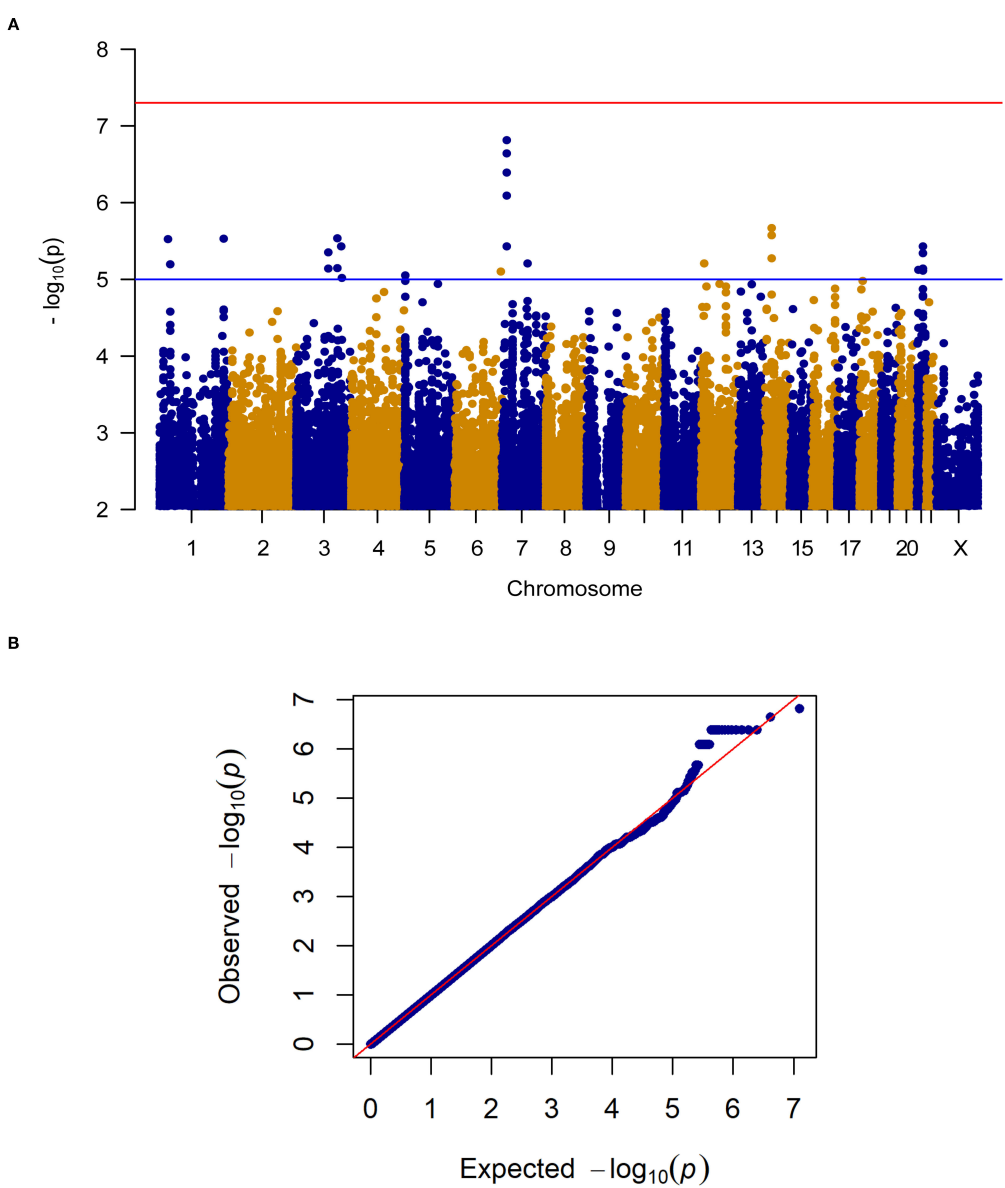
**(A)** Manhattan plot of GWAS of change in pain severity among 212 IBS patients controlling for age, sex, treatment arm and the first 5 principal components for genetic ancestry. The x-axis is chromosome position and the y-axis is *p*-value. The red line represents the “genome-wide significant” threshold, 5.0E-8, and the blue line denotes the “genome-wide suggestive” threshold, 1.0E-5. We did not find genome-wide significant SNPs for change in pain severity, but we did find a few genome-wide suggestive SNPs. **(B)** Quantile-quantile plot of the data. The genomic inflation factor λ is 0.994, which indicates that no inflation of data was observed in the GWAS of change in pain severity from baseline to 6 weeks across all treatment arms.

When considering change in pain severity from baseline to endpoint of the trial, no SNPs reached the genome-wide significance threshold (*P* < 5.0E-8) however, 24 SNPs in close proximity on Chromosome 7 (Chr7: 17,705,199–17,710,866) reached genome-wide suggestive significance (*P* < 1.0E-5; see Figure 2 for the Manhattan and quantile-quantile plot and Supplementary Table 1 for SNPs). These loci on Chromosome 7 are proximal to the gene for SNX13, associated with intracellular trafficking. Interestingly, this genomic region has been associated with chronic widespread pain previously (17), where it was suggested to be linked to a reduced biodiversity of the gut microbiome. SNPs in this region also reach genome-wide suggestive *P*-values in the female patient only sensitivity analysis. After treatment, met alleles in SNP rs1105794 were associated with decreased overall pain severity while val alleles were associated with increased overall pain severity. At baseline, met alleles in rs1105794 were associated with greater pain frequency (*P* < 1.0E-3) and a minimal decrease in pain severity.

**Figure 3:**
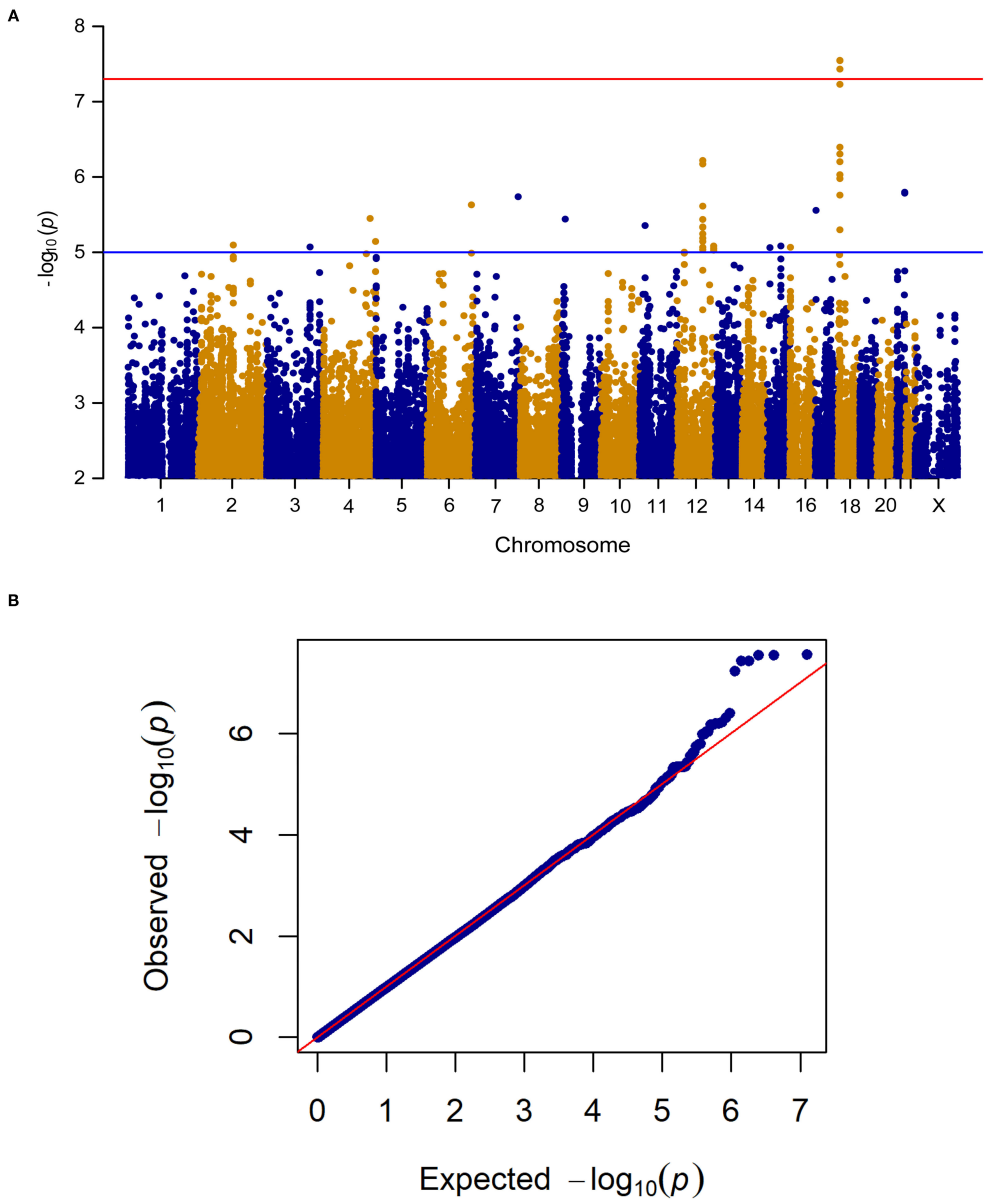
**(A)** Manhattan plot of GWAS of change in pain frequency among 212 IBS patients controlling for age, sex, treatment arm and the first 5 principal components for genetic ancestry. The x-axis is chromosome position and the y-axis is *p*-value. The red line represents the “genome-wide significant” threshold, 5.0E-8, and the blue line denotes the “genome-wide suggestive” threshold, 1.0E-5. We find five genome-wide significant SNPs rs1105794, rs4479336, rs6506387, rs4798443, rs9952528, and a few genome-wide suggestive SNPs. **(B)** Quantile-quantile plot of the data. The genomic inflation factor λ is 0.989, which indicates that no inflation of data was observed in the GWAS of change in pain frequency from baseline to 6 weeks across all treatment arms.

When analyzing change in pain frequency over the 6 week treatment trial, five SNPs (rs1105794, rs4479336, rs6506387, rs4798443, rs9952528) within close proximity on chromosome 18 reached genome-wide significance (see Figure 3 for the Manhattan and quantile-quantile plot and Supplementary Table 2 for SNPs). The same SNPs were found to be genome-wide significant in the women-only cohort. An additional nine SNPs within this region of chromosome 18 (Chr18: 6,451,334–6,460,576) were of genome-wide suggestive significance.

These SNPs mapped closely to *L3MBTL4* which encodes the histone methyl-lysine binding protein and was genome-wide significant in a GWAS of pain severity in dysmenorrhea (13), a pain syndrome in women characterized by pain with menses.

### *Post-hoc* Analysis of Findings

**Figure 4:**
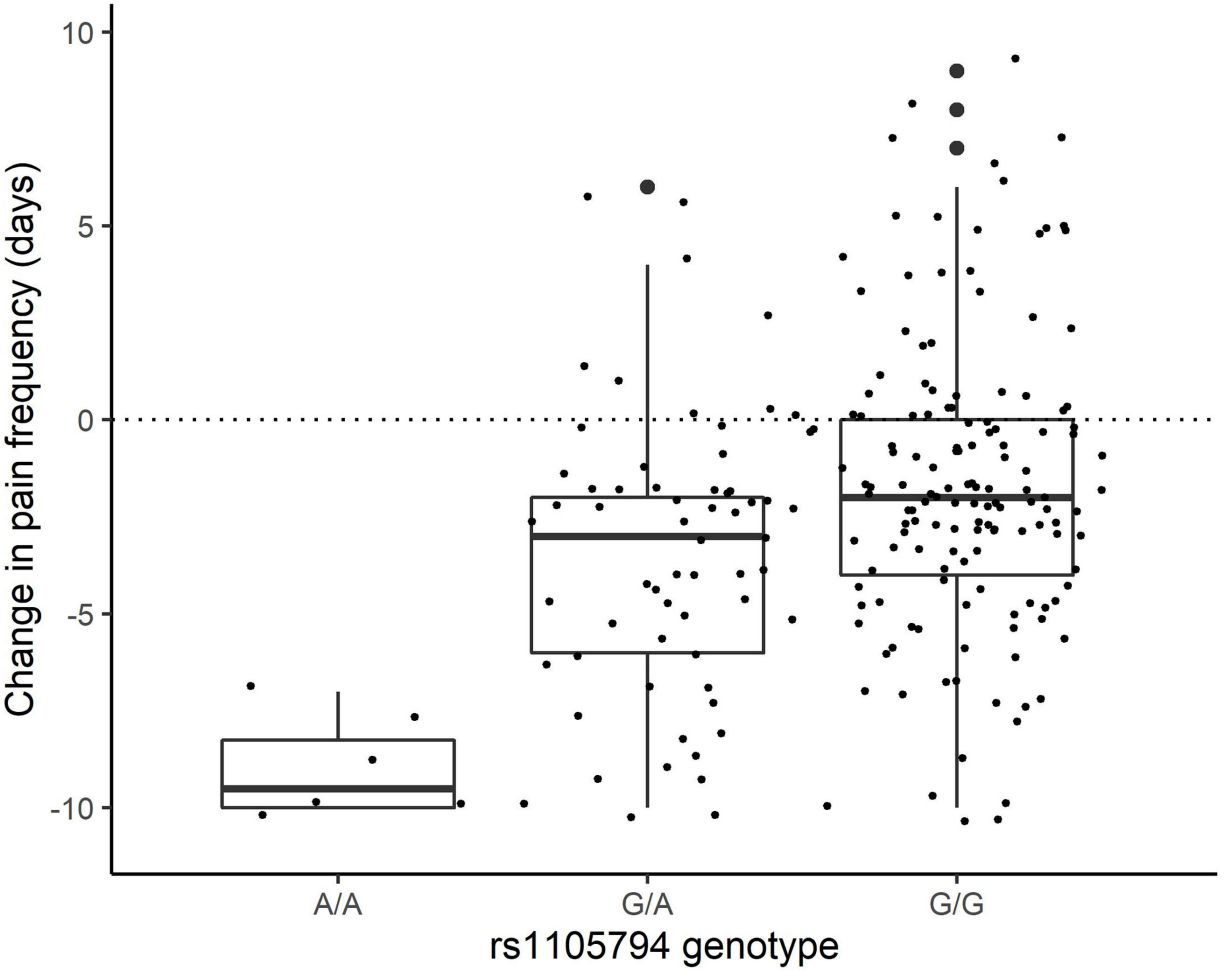
Change in pain frequency by rs1105794 across three allele variants: met/met (A/A), val/met (G/A), val/val (G/G).

For change in pain frequency, rs1105794 showed a large significant effect (measured as change in days of pan) of 2.86 (95% CI 1.88–3.84; *p* < 0.0001) and followed a dose-response model. The difference between G/G and G/A equaled an effect size of 0.46, between G/A and A/A an effect size of 1.44, and between the homozygous variants the effect size was 1.90 (see Figure 4). Most of this effect was mediated directly by rs1105794, so although present, the indirect effect mediated by estradiol was small (−0.12, 95% CI −0.08, −0.45; *P* = 0.383).

rs1105794 did not influence estradiol change (*P* = 0.3521). The result was similar for estrone and did not change in quality if only women were included or sex was treated as a covariate.

## Discussion

Here we report the findings of a candidate gene analysis, an exploratory GWAS of pain frequency and an exploratory GWAS of pain severity in a RCT of 4 different IBS treatments [double-blind peppermint oil (DBM), double-blind placebo (DBP), open-label (OLP), and a no placebo pill control (NPC)] (10). The candidate gene analysis showed a significant effect of *COMT* rs4680 on change in pain severity but not change in pain frequency. In an exploratory GWAS, the lead SNPs for change in pain frequency were genome-wide significant and mapped to a region on chromosome 18 proximal to *L3MBTL4* whereas the lead SNPs associated with pain severity were genome-wide suggestive and mapped to a region on chromosome 7 proximal to *SNX13*.

We have previously found that *COMT* rs4680 genotype was associated with improvement in IBS symptoms as measured by IBS-SSS (14). In this study, we aimed to further investigate whether *COMT* rs4680 genotype was associated with specific IBS symptoms, particularly pain severity and pain frequency. *COMT* is an enzyme that metabolizes endogenous catechols, including estrogen, dopamine, norepinephrine, and epinephrine. Due to its role as a key regulator of dopamine in the prefrontal cortex, a brain region that processes pain signaling, COMT has been investigated extensively in the context of depression and Parkinson's disease. Our SNP of interest, *COMT* rs4680, encodes a transversion of G-to-A, which results in a substitution of methionine (met) in place of valine (val) and a 3-to-4 fold reduction in enzymatic activity (15, 16). In our previous findings, increasing met alleles corresponded to stronger placebo response (14). *COMT* rs4680 genotype has been shown to be associated with pain sensitivity in patients treated with morphine after cardiac surgery (18), and with pain severity in Parkinson's disease patients (19) and hospitalized burn victims (20). Specifically, those with the low activity A allele (which corresponds to met) had higher pain sensitivity. Importantly, the *COMT* rs4680 met allele substitution has also been shown to be associated with placebo analgesia specifically (21, 22). Our finding that *COMT* rs4680 met is associated with a decrease in pain severity in IBS patients aligns with prior findings on the role of *COMT* in pain mediation.

Our finding that pain frequency scores are related to SNPs that are close to the L3MBTL4 gene on chromosome 18 is a novel finding that has basis in the literature. *L3MBTL4* encodes histone methyl-lysine binding protein that is predicted to be involved in negative regulation of transcription and is highly expressed in gonadal tissue. *L3MBTL4* was nominally genome-wide significant in a GWAS of pain severity in dysmenorrhea in Japanese women. In our study, the lead SNP (rs1105794), had a minor allele frequency of 18%. This study was small and exploratory, but findings of the juxtaposition of pain phenotypes in IBS and dysmenorrhea in women warrant replication studies to confirm the potential for SNPs in this locus to impact change in IBS pain frequency, particularly as women with IBS have higher rates of dysmenorrhea than the general population (21, 23). Symptoms of IBS are two times as prevalent in women than in men (22, 24). Further, female patients with IBS tend to report increased symptoms during menstruation (23–26). In Western countries, women demonstrate a greater prevalence of IBS, and are more likely to seek treatment for the disorder than men (25, 27). Among the sex differences in the presentation of IBS, women more frequently endorse constipation, abdominal distention, and extraintestinal visceral symptoms such as muscle stiffness (26, 28). Several studies have reported on these sex differences in the context of IBS, however, the mechanisms mediating their presentation are not well defined. In recent years, increased focus has been placed on the role of hormones in modulating sex differences in the presentation of functional gastrointestinal disorders (27, 29). Together with our findings, these studies indicate the potential for identification of the cause of sex-linked symptom profiles, particularly in conditions associated with chronic pain.

In contrast, changes in pain severity were related to *SNX13* which encodes a protein that contains both a phosphoinositide binding domain and a regulator of G protein signaling domain. Overexpression of the protein that this gene encodes is associated with delayed degradation of epidermal growth factor (EGF) receptor (30). EGF activity was linked to visceral hypersensitivity in an IBS rodent model (31). Further, EGF activity is associated with neuropathic pain (30, 32) and chronic pain processing (31, 33). Importantly, *SNX13* has previously been associated with widespread pain in patients with IBS (17). Though many of the findings in the literature are tangential to nociplastic pain and IBS specifically, our findings point to the importance of distinguishing between different elements of the IBS pain profile to better understand patient symptoms and the underlying genetic loci.

This study is the first to consider genetic associations with IBS-SSS subscores. The IBS-SSS is a very useful tool used in the diagnosis and management of IBS symptoms, but the subscore components vary significantly in scope. This reflects IBS as a complex condition with varying phenotypic elements, each of which is likely related to specific genetic loci. Utilizing the aggregate score could minimize inter-patient differences and obscure the benefit of treatments which target a particular subcomponent of IBS-SSS. In order to move toward more efficacious and precise treatment for patients with IBS, thorough investigation of the genetic underpinnings of each subscore in a larger sample size is necessary.

## Data Availability Statement

The original contributions presented in the study are included in the article/Supplementary Materials, further inquiries can be directed to the corresponding author/s.

## Ethics Statement

The studies involving human participants were reviewed and approved by the Ethics Review Board at Beth Israel Deaconess Medical Center under protocol 2015P000282. The patients/participants provided their written informed consent to participate in this study.

## Author Contributions

KH, AL, TK, JL, JS, and VC contributed to project design and analysis, IBS trial design and execution (JI and JN). In addition: JV conducted statistical analysis and drafted the manuscript. RW conducted genetic analyses and drafted the manuscript. JS, SR, and HY contributed to writing the final manuscript. All authors read, commented on, contributed to and approved the final version of the manuscript.

## Funding

This work was supported, in part, by: NIH grant R01AT008573 to AL and TK; NIH grants HL119145, HG007690, and GM107618; AHA grants D007382 and CV-19, and Rockefeller Foundation grant A015025 to JL; NIH K23 DK119584 to JS; and NIH K01HL130625 to KH. KH and HY were funded by a pilot grant from the Osher Center for Integrative Medicine at Brigham and Women's Hospital and Harvard Medical School.

## Conflict of Interest

AL owns stock in Bristol Myer Squibb and Johnson & Johnson and receives income from Vibrant and Mylan. JL is the scientific co-founder of Scipher Medicine, Inc. JN consults for World Clinical Care. JS has received consultancy fees from Alimentiv, Mozart Therapeutics, Takeda Pharmaceuticals, and Teva Pharmaceuticals. JV has received consultancy fees from Vertex Pharmaceuticals and Embody Orthopedics outside the submitted work. The remaining authors declare that the research was conducted in the absence of any commercial or financial relationships that could be construed as a potential conflict of interest.

## Publisher's Note

All claims expressed in this article are solely those of the authors and do not necessarily represent those of their affiliated organizations, or those of the publisher, the editors and the reviewers. Any product that may be evaluated in this article, or claim that may be made by its manufacturer, is not guaranteed or endorsed by the publisher.

## Abbreviations

COMT: Catechol-O-methyltransferase
DBP: Double blind placebo
DBM: Double blind mint
IBS-SSS: IBS symptom severity scale
OLP: Open label placebo
NPC: No-pill control
GWAS: Genome-wide association study.
